# Net Assimilation Rate and Agronomic Efficiency of Nitrogen in Tartago (*Ricinus communis* L.) (Euphorbiaceae) in Dry Climate

**DOI:** 10.1155/2020/7064745

**Published:** 2020-08-21

**Authors:** Ernesto Díaz-López, Jesús M. E. Aguilar-Luna, Juan M. Loeza-Corte

**Affiliations:** ^1^Ingeniería en Agricultura Sustentable y Protegida, Universidad Tecnológica de Tehuacán, San Pablo Tepetzingo Tehuacán, Puebla 75859, Mexico; ^2^Ingeniería Agroforestal, Complejo Regional Norte–Sede Tetela, Benemérita Universidad Autónoma de Puebla, Tetela de Ocampo, Puebla 73640, Mexico; ^3^Ingeniería en Agroindustrias, Instituto de Tecnología de Los Alimentos, Universidad de La Cañada, Teotitlán de Flores Magón, Oaxaca 68540, Mexico

## Abstract

To know the dynamics of net assimilation rate and the agronomic efficiency of nitrogen in the Tartago crop, seeds of three accessions were collected in Teotitlán de Flores Magón, Oaxaca, Mexico. The treatments consisted of nitrogen fertilization of 0, 20, 40, 60, 80, 100, 120, and 140 kg (N) ha^−1^, evaluated under a completely randomized design. The experimental unit was constituted by a Tartago plant inside of a polyethylene bag with soil of the zone, and four repetitions were considered. The response variables were dry biomass, number of fruits per plant, agronomic yield, harvest index, nitrogen agronomic efficiency, SPAD units, and net assimilation rate. The results indicate that climatic conditions did not influence the growth and development of the crop. The maximum values for all of response variables were achieved with the application of nitrogen in a range of 60 to 140 kg ha^−1^. The net assimilation rate was adjusted to a quadratic model. It is concluded that the Tartago responds positively to the application of nitrogen and can be an alternative to be grown in dry climate.

## 1. Introduction

Tartago (*Ricinus communis* L.) is a perennial plant that belongs to the Euphorbiaceae family [1]. Its origin center is tropical Africa [2]. For many years, its seeds were used for the extraction of drying oil used in the manufacture of paints and cosmetics [3, 4] and currently in the synthesis of biofuels with the help of microorganisms such as *Pseudomonas* and *Candida* spp. [5]. Physiologically, for its large size, the Tartago has a high photosaturation point; due to this, it presents great adaptability to dry areas, such as the Tehuacán-Cuicatlán Valley. For the above, the Tartago grows wild to the edge of cultivated fields, supporting radiations greater than 900 W m^−2^ that other plants would not support, converting it into an alternative crop that will withstand the environmental conditions of the area, and from this way, there is an opportunity for the cultivation of this plant by the inhabitants of the mentioned valley. Regarding the net assimilation rate, this is a physiological index that allows us to know the amount of biomass accumulated by the plant per unit of leaf area at a given time (g cm^−2^ day^−1^) [6, 7]. The importance of this index lies in providing information on the behavior of the plant's photosynthetic machinery, being an indirect measurement of photosynthesis, which can be related to the accumulated biomass in the organ of interest of the plant and, thus, correlate the behavior of a certain genotype with a given environment [8]. On the other hand, the importance of nitrogen as a fertilizer is known, it increases growth and higher biomass yields, and it affects the proportion of amino acids lysine and threonine; in oilseeds, protein content increases but has an adverse effect on oil content; it is used to maintain high production levels of quantity and quality [9]. Thus, under this trend [10], they mention that when the nitrogen content in the soil is not known, fertilizer doses higher than those required by the crop are applied, causing intoxication. Other authors cite that, for nitrogen, in soils suitable for agriculture in tropical areas, they present severe deficiencies and low availability of this, and, therefore, it is important to carry out a soil analysis before planting a crop [11]. In relation to the agronomic efficiency of nitrogen, it provides information to know how efficient a given genotype is to assimilate this nutrient and transform it into dry biomass [12] and thus provide the necessary nutrient to the crop, without applying excess nitrogen, which can be lost by leaching or sublimation, and become a further contaminant of the atmosphere and groundwater in areas of intensive agriculture [13]. Therefore, the main objective of this research was to evaluate the net assimilation rate and agronomic efficiency of nitrogen of eight levels of nutrients in the culture of Tartago. The derived hypothesis was that the nitrogen applied in different doses will affect the net assimilation rate as well as the efficiency in the use of nitrogen in the crop of Tartago, when sown under dry climate conditions.

## 2. Materials and Methods

### 2.1. Location of the Experiment

The present study was carried out in Teotitlán de Flores Magón, Oaxaca, located at 18° 08′ latitude north, 97° 05′ longitude west, and 888 m of the altitude above sea level. Under the climatic classification of Köppen modified for García [14], the crop was developed under a climate Bs_1_(w') (h') heg, which corresponds to a dry climate with an annual average temperature higher than 18°C and lower than 27°C. Precipitation is greater than 200 mm and less than 600 mm, whose distribution goes from June to September with the presence of intraestival drought. The temperature oscillation is greater than 7°C and less than 14°C between the warmest and coldest months; the warmest month occurs before the summer solstice, this being April.

### 2.2. Germplasm

The germplasm was obtained from three accessions of Tartago (*Ricinus communis* L.), because they dominate in the area; these were collected in the town of San Antonio Nanahuatipam, Oaxaca, municipality of Teotitlán de Flores Magón at 18° 07′ 00″ latitude north, 97° 04′ 00″ longitude west, and 795 m of the altitude above sea level, whose taxonomic identification was made with the specific keys for Euphorbiaceae [15, 16].

### 2.3. Sowing and Crop Management

The selected seeds were planted in polyethylene bags with a capacity of 4 kg. Each bag contained soil of the zone (which corresponds to a luvisol in formation process) with colluvial remnants mixed with mesquite leaf litter (*Prosopis* spp.) at a 2 : 1 (v/v) ratio. The pH and electrical conductivity were 7.8 and 2.7 dS m^−1^, respectively. These were measured after homogenizing the soil and before filling the bags with the soil mixture. In both cases, a homogeneous soil sample was taken and suspended in deionized water at a 1 : 2.5 soil-to-water ratio (modified [17]. The initial nitrogen content was 4.3 mg kg^−1^, which was determined by the Kjeldahl method [18]. The organic matter present was 3.1%; it was determined by wet chemical oxidation with 1 M potassium dichromate [19]. The plants together with the bag were placed under a topological arrangement of 0.50 × 0.80 giving a total of 2,500 plants ha^−1^. For weed control, a manual weeding was performed weekly.

### 2.4. Design Experimental Unit and Treatments

The experimental design was completely randomized according to

(1) $$\begin{array}{c} y_{ij}=\mu +\tau_{i}+\varepsilon_{ij}, \end{array}$$

where *y*_*ij*_ is the response variable of the *i*-th level of nitrogen in the *j*-th repetition, *μ* is the true overall mean, *τ*_*i*_ is the effect of the *i*-th nitrogen treatment, and *ε*_*ij*_ is the experimental error of the *i*-th level of nitrogen in the *j*-th repetition [20]. The treatments were eight levels of nitrogen: 0, 20, 40, 60, 80, 100, 120, and 140 kg ha^−1^ and four repetitions (8 × 4) = 32 experimental units. The fertilization formula was supplemented with 50 kg ha^−1^ of phosphorus and 20 kg ha^−1^ of potassium. The sources of these nutrients were urea (46% N), triple calcium superphosphate (46% P_2_O_5_), and potassium chloride (60% K_2_O). The experimental unit was constituted by a bag of polyethylene plus the substrate and the Tartago plant.

### 2.5. Response Variables

For dry biomass, it was determined by drying the stem, leaves, and pericarp in a forced convection oven at 70°C until reaching a constant weight [7]. The number of fruits per plant, the number of true fruits containing seeds, was counted before dehiscence. For agronomic yield, the weight of the botanical seeds produced per plant was determined using a Sartorius Analytical Balance model TE601, and the result was expressed in g plant^−1^. For the harvest index, it was determined by

(2) $$\begin{array}{c} \text{HI}=\frac{\text{AY}}{\text{BY}}, \end{array}$$

where HI is the harvest index, AY is the agronomic yield, and BY is the biological yield. The Agronomic Efficiency of Nitrogen was determined using

(3) $$\begin{array}{c} \text{AEN}=\frac{{\text{AY}}_{+N}-{\text{AY}}_{-N}}{N_{A}}, \end{array}$$

where AEN is the agronomic efficiency of nitrogen (kg of seed per kg of nitrogen applied per m^−2^), AY_+*N*_ is the agronomic yield whit nitrogen, AY_−*N*_ is the agronomic yield without nitrogen, and *N*_*A*_ is the nitrogen applied [3]. For SPAD units, these were evaluated with the chlorophyll meter Minolta-502, taking the reading on five sheets, to obtain the respective average [21], taking the reading directly on the leaf at intervals of 30 days. The net assimilation rate was determined by

(4) $$\begin{array}{c} \text{NAR}=\left(\frac{W_{2}-W_{1}}{T_{2}-T_{1}}\right)\left(\frac{\ln\;LA_{2}-\ln\;LA_{1}}{LA_{2}-LA_{1}}\right), \end{array}$$

where NAR is the net assimilation rate, *W*_2_ and *W*_1_ are the dry biomass weights of the plant at the respective time *T*_2_ and *T*_1_, and LA_2_ and LA_1_ are the respective leaf areas corresponding to the time *T*_2_ and *T*_1_ [7]. The leaf area was determined by triangulation of the leaf, dividing the whole leaf in triangles and, later, adding the areas of each leaf to obtain the total area [22]. When the response variables were found to be significant, Tukey's multiple comparison test was applied at a significance level of 0.05.

## 3. Results and Discussion

### 3.1. Weather Conditions

**Figure 1:**
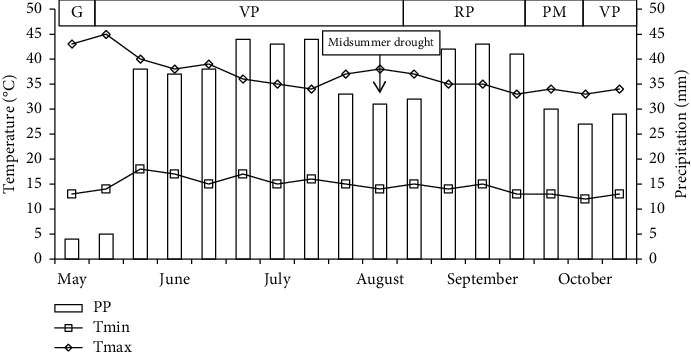
Decennial average of precipitation and both maximum and minimum temperature during the development of the Tartago crop (*Ricinus communis* L.), in dry weather at Teotitlán de Flores Magón, Oaxaca, Mexico, in 2018. G: germination; VP, vegetative phase; RP, reproductive phase; PM, physiological maturity.

The temperature and precipitation conditions, as well as the phenology recorded during the Tartago cultivation cycle, are shown in Figure 1. Ten-day averages are presented for precipitation and both maximum and minimum temperature values. The maximum temperature ranged between 45°C and 34°C, presenting the maximum value at the end of May. The minimum temperature was distributed in a range of 18°C to 13°C. The total precipitation during the ontogenetic cycle was 561 mm, and this was distributed from May to October. The maximum precipitation occurred during July with 131 mm (23.3%). The presence of midsummer drought was observed in August. It is worth mentioning that under these climatic conditions, the Tartago crop was developed without problems until reaching physiological maturity.

### 3.2. Response Variables

Analysis of variance and the multiple comparison test are presented in Table 1. It can be seen that there were highly significant differences for all variables, except for the harvest index, which turned out to be nonsignificant. The coefficient of variation oscillated between 6.23 and 25.25%, showing that the data were reliable. Regarding biomass, treatments of 60, 80, 100, 120, and 140 kg(N) ha^−1^ were found to be statistically equal, although there were numerical differences and it was in these, where the maximum values were reached. The higher agronomic yield was achieved by applying nitrogen in a range of 40 to 140 kg(N) ha^−1^, and the treatments mentioned were also statistically equal. In this way, the maximum seed yield was 81.33 g plant^−1^, which was the result of applying 100 kg(N) ha^−1^, while the lower yield of seed was for the control treatment and 20 kg(N) ha^−1^, with 57.12 and 60.55 g plant^−1^, respectively. These results differ with those obtained by Hernández et al. [23], who worked with the EN-16 variety in the Guanajuato shallows and obtained a yield of 109.20 g plant^−1^, 34.26% more than that in this study. This difference is due to the heterogeneity existing between the genotypes of both studies, in addition to the environmental differences of each zone, which affect the expression of the phenotype of both materials. In the same way, as in biomass, the highest number of fruits was reached in the range of 60 to 140 kg(N) ha^−1^, while the control and treatments 20 and 40 kg(N) ha^−1^ presented the lowest number of fruits with 6.33, 8.44, and 9.00 fruits per plant. The agronomic efficiency of nitrogen in the Tartago cultivation indicated that the maximum efficiency of this element occurred when applying 100 kg(N) ha^−1^, achieving an accumulation of 0.26 kg of biomass per kg of nitrogen applied in the Tartago plant, while the control treatment only reached an efficiency of 0.02 kg (N) plant^−1^. The previous response could be due to the fact that the germplasm used presents a high genetic variability, causing few responses to the application of nitrogen. This suggests applying doses greater than 140 kg(N) ha^−1^. The above data differ from the study by Rico et al. [24], who report that the yield of Tartago seed in Michoacán, Mexico, was 260 g plant^−1^ with an agronomic efficiency of nitrogen of 0.30 kg(N) applied. These differences could be attributed to the different population densities used for each study.

### 3.3. Net Assimilation Rate

**Figure 2:**
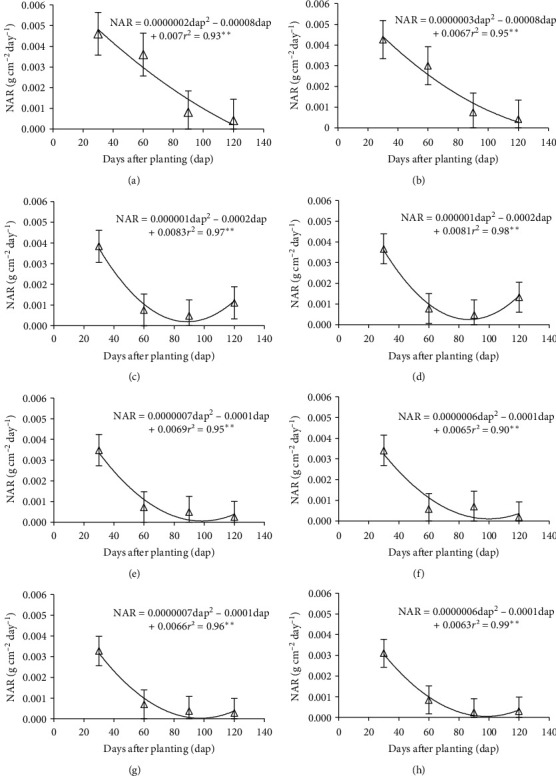
Dynamics of the net assimilation rate in Tartago (*Ricinus communis* L.) in the function of four levels of nitrogen. (a) 0 kg(N) ha^−1^; (b) 20 kg(N) ha^−1^; (c) 40 kg(N) ha^−1^; (d) 60 kg(N) ha^−1^; (e) 80 kg(N) ha^−1^; (f) 100 kg(N) ha^−1^; (g) 120 kg(N) ha^−1^ in Teotitlán de Flores Magón, Oaxaca, Mexico, in 2018. NAR, net assimilation rate.

The net assimilation rate for levels 0 and 20 kg(N) ha^−1^ was fitted to a quadratic model descending from 30 to 120 days after planting (dap), decreasing from 0.0049 to 0.00042 g cm^−2^ day^−1^ (Figure 2). On the other hand, high levels of nitrogen in 80, 100, 120, and 140 kg(N) ha^−1^ showed a decreasing behavior (Figures 2(e)to 2(f)), on average 30 to 90 dap from 0.0032 to 0.00025 g cm^−2^ day^−1^. This indicates that as the plant of Tartago develops, the NAR tends to decrease with respect to time. This has been proven by Aguilar et al. [25], who studied NAR in sunflower cultivation in the function of the population density and mention that it tends to decrease with respect to time, due to the senescence of basal leaves, the phenomenon that also happens in the Tartago in spite of having different species to be cultivated, under contrasting ecological conditions.

### 3.4. SPAD Units

**Figure 3:**
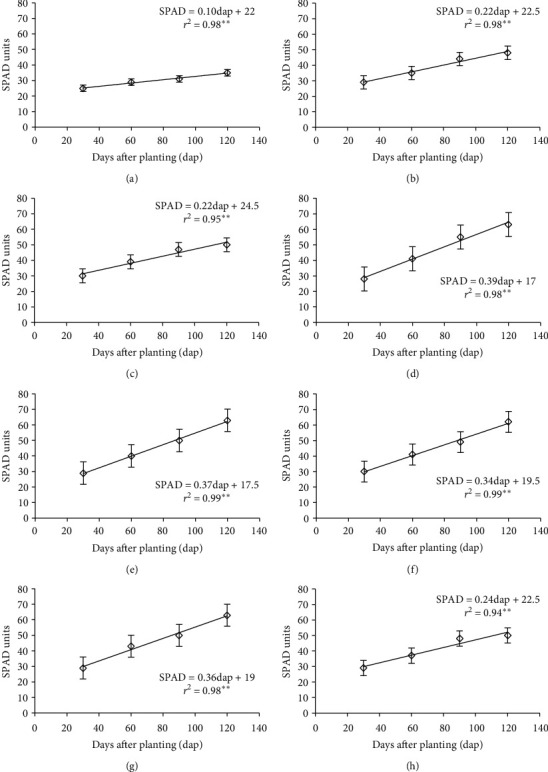
SPAD units in the culture of Tartago (*Ricinus communis* L.), in function of four levels of nitrogen. (a) 0 kg(N) ha^−1^; (b) 20 kg(N) ha^−1^; (c) 40 kg(N) ha^−1^; (d) 60 kg(N) ha^−1^; (e) 80 kg(N) ha^−1^; (f) 100 kg(N) ha^−1^; (g) 120 kg(N) ha^−1^, in Teotitlán de Flores Magón, Oaxaca, Mexico, in 2018.

In all levels of nitrogen, their determination coefficients were highly significant, being between 0.94 and 0.99, which indicates that 94 to 99% of SPAD units were explained by the increase in nitrogen fertilization. Between the remaining 6 and 1%, it is explained by other variables. In mathematical models, SPAD units increase as the amount of nitrogen applied increases (Figure 3). These results coincide with those reported by Cano et al. [26], who applied nitrogen to *Arundo donax*; they mention that the chlorophyll content increases as nitrogen fertilization increases. At a dose of 80, 100, and 120 kg(N) ha^−1^, the slope of the curve was 0.37, 0.34, and 0.36, respectively, finding a variation between them of 0.03 units, a range that turns out to be very small. Regarding the highest dose of nitrogen, the slope decreased considerably to 0.24, finding a variation with respect to the doses of previous nitrogen of 0.13 units. This last slope resembles the low levels of this nutrient in 20 kg(N) ha^−1^ (Figure 3(b)), which presented a slope of 0.22; there is a difference between them of 0.02 units. This indicates that the crop responds negatively to the upper application of 140 kg(N) ha^−1^. This fact was reflected equally in the agronomic yield, as it was not significant between the application of 120 and 140 kg(N) ha^−1^. The above results indicate that by increasing nitrogen from 20 to 120 kg(N) ha^−1^, the SPAD units increase, thus becoming a good indicator of the nutritional status of the plant with respect to nitrogen. The SPAD units of this study coincide with those reported by Rincón and Ligarreto [27], who, when applying 150 kg(N) ha^−1^, report values of 50 SPAD units, and in low doses of nitrogen, they reported 30 SPAD units, concluding that the SPAD units can be an excellent estimator to determine the nitrogen content in the maize crop.

## 4. Conclusions

The net assimilation rate in Tartago was adjusted to descending quadratic models at all levels of nitrogen applied. The highest agronomic efficiency of nitrogen was achieved with the application of 60, 80, 100, and 120 kg(N) ha^−1^. The highest agronomic yield in Tartago was obtained with the application from 40 to 140 kg(N) ha^−1^. SPAD units can be an excellent estimate of the nutritional status in plants of Tartago, with respect to nitrogen. Due to its broad phenotypic plasticity, tartar cultivation can be a reliable alternative for growing under dry climate conditions.

## Figures and Tables

**Table 1:** Multiple comparison test in Tartago (*Ricinus communis* L.) for five variables in response to the application of eight levels of nitrogen in Teotitlán de Flores Magón, Oaxaca, Mexico, in 2018.

Treatment, kg (N) ha^−1^	B	AY	NF	HI	AEN
	g plant^−1^				kg kg(N) plant^−1^
0	310.11 c^¶^	57.12 b^¶^	6.33 b^¶^	0.18 a^¶^	0.02 c^¶^
20	312.82 c	60.55 b	8.44 b	0.19 a	0.17 b
40	389.22 b	73.12 a	9.00 b	0.18 a	0.18 b
60	410.50 a	78.75 a	12.89 a	0.19 a	0.22 a
80	421.60 a	80.27 a	13.44 a	0.19 a	0.25 a
100	432.80 a	81.33 a	14.74 a	0.18 a	0.26 a
120	450.80 a	71.00 a	14.99 a	0.16 a	0.22 a
140	440.50 a	71.33 a	16.00 a	0.16 a	0.20 b

SDH	40.90^*∗∗*^	13.50^*∗∗*^	4.50^*∗∗*^	0.02 ns	0.04^*∗∗*^
CV%	22.12	19.33	25.25	6.23	15.89

B, biomass; AY, agronomic yield; NF, number of fruits; HI, harvest index; AEN, agronomic efficiency of nitrogen; SDH, significant difference honest; CV; coefficient of variation; n.s., not significant. ^¶^Values within columns with the same alphabets are statistically the same according to Tukey at *P* ≤ 0.05. ^*∗∗*^ and ^*∗*^ indicate significance at 0.01 and not significant, respectively.

## Data Availability

The data used to support the findings of this study are available from the corresponding author upon request.

## References

[1] Jachmanian I., Pérez G. E., Villamil J. (2009). The cultivation of tártago (*Ricinus communis* L.) in Uruguay. *Boletín de Divulgación. INIA.*.

[2] Barbona A. S. (2003). The production of Tartago a sustainable crop and income for the north of Argentina. *National Institute of Agricultural Technology*.

[3] Díaz L. E., Orlando G. I. J., Campos M. J. M., Brena H. I., Loeza C. J. M. (2013). Castor oil (*Ricinus communis* L.) with application in optical communications. *Ciencias Agrícolas Informa*.

[4] Mazzani E., Rodríguez E. (2009). Study of the variability present in germplasm of tártago (*Ricinus communis* L.) in relation to clusters, fruits and seeds. *Revista UDO Agrícola*.

[5] Castaño C. L., Torres M. J. A., Cardona A. C. A., Orrego C. E. (2004). Production of biodiesel from vegetable oils using free enzymes: preliminary study. *Ingeniería de recursos naturales y del ambiente*.

[6] Carpio C. A., Escalante E. J. A. S., Aguilar M. I. (2016). Analysis of growth and yield of maize in warm climate according to genotype, biofertilizer and nitrogen. *Terra Latinoamericana*.

[7] Escalante E. J. A. S., Kohashi S. J. (2014). *Bean Yield and Growth. Manual for Data Collection*.

[8] Mora A. R., Ortíz‐Cereceres J., Rivera‐Peña A., Mendoza‐Castillo M. C., Colinas‐León M.T., Lozoya‐Saldaña H. (2006). Efficiency indexes of potato genotypes established under rained conditions. *Revista Chapingo Serie Horticultura*.

[9] Maheswari M., Murthy A. N. G., Shanker A. K., Abrol Y. P., Adhya T. K., Singh (2017). Nitrogen nutrition in crops and its importance in crop quality. *The Indian Nitrogen Assessment. Sources of Reactive Nitrogen, Environmental and Climate Effects, Management Options and Policies. Section B: Nitrogen Processes in the Biosphere*.

[10] Villareal R. M., García E. R. S., Osuna E. T., Armenta B. A. D. (2002). Effect of dose and nitrogen source on yield and postharvest quality of tomato in fertigation. *Terra Latinoamericana*.

[11] Sosa R. B. A., Garcia V. Y. S. (2018). Efficiency in the use of nitrogen in organically and mineral fertilized corn. *Agronomia Mesoamericana*.

[12] Pichardo R. J. C., Escalante J. A., Rodríguez M. T., Sánchez G G. P. (2007). Divided application and agronomic efficiency of nitrogen, water and radiation use and bean yield. *TERRA Latinoamericana*.

[13] Pacheco A. J., Cabresa S. A. (2003). Main sources of nitrogen in groundwater. *Ingeniería, Revista Académica*.

[14] García E. (2004). *Modificaciones al Sistema de Clasificación Climática de Köppen, Serie Libros, núm. 6, Instituto de Geografía*.

[15] Martínez G. M., Jiménez R. J., Cruz D. R. (2002). The genera of the family Euphorbiaceae in Mexico. *Anales del Instituto de Biología. Serie Botánica. Universidad Nacional Autónoma de México*.

[16] Steinmann V. W. (2002). Diversidad y endemismo de la familia Euphorbiaceae en Mexico. *Acta Botanica Mexicana*.

[17] Van Zwieten L., Kimber S., Morris S. (2010). Effects of biochar from slow pyrolysis of papermill waste on agronomic performance and soil fertility. *Plant and Soil*.

[18] Kjeldahl J. (1883). Neue Methode zur Bestimmung des Stickstoffs in organischen Körpern. *Fresenius’ Zeitschrift für analytische Chemie*.

[19] Walkley A., Black I. A. (1934). An examination of the degtjareff method for determining soil organic matter, and a proposed modification of the chromic acid titration method. *Soil Science*.

[20] Cochran G. W., Cox M. G. (1990). Experimental designs. *Trillas*.

[21] Martínez G. L., Reyes G. Y., Falcón R. A., Napoles G. M. C., Nuñez V. M. C. (2016). Effect of bioactive products on biofertilized bean (*Phaseolus vulgaris* L.) plants. *Cultivos Tropicales*.

[22] Arteaga F. (2012). Cálculo del área de un polígono simple. *Modelling in Science Education and Learning*.

[23] Hernández M. M., Zamarripa C. A., Teniente O. R., González A. A., Solís B. J. L. (2012). Technical guide for the production of higuerilla (*Ricinus communis* L.) in guanajuato. Instituto nacional de Investigaciones agrícolas y pecuarias. *Folleto Técnico*.

[24] Rico P. H., tapia V. L., Teniente O. R. (2011). Guide for growing higuerilla (*Ricinus communis* L.). *Michoacán. Boletín Técnico 1*.

[25] Aguilar G. L., Escalante E. J. A., Fucikovsky Z. L., Tijerina Ch. L., Engleman C. E. M. (2005). Leaf area, net assimilation rate, yield and population density in sunflower. *Terra Latinoamericana*.

[26] Cano R. J., Sanz M., Curt M. D., Plaza A., Lobo M. C., Mauri P. V. (2020). *Fertigation of Arundo donax L. With Different Nitrogen Rates for Biomass Production*.

[27] Rincón C. A., Ligarreto G. A. (2010). Relationship between leaf nitrogen and chlorophyll content in maize associated with pastures in the Colombian foothill piedmont. *Corpoica. Ciencia Y Tecnología Agropecuaria*.

